# Supplementary material for: Diagnostic Reference Levels in pediatric interventional cardiology: A multicenter study by the French cohort in HARMONIC project
Source: PLoS One. 2025 Sep 17;20(9):e0332241. doi: 10.1371/journal.pone.0332241 (PMC12443263; doi:10.1371/journal.pone.0332241)
Supplement: Attachment — Submitted filename: PLOS review.docx [file pone.0332241.s001.docx]

**Reviewer -1 Comments on Manuscript PONE-D-25-10735**

**Title:** Diagnostic Reference Levels in Pediatric Interventional Cardiology: A Multicenter Study by the French Cohort in HARMONIC Project
**Journal:** PLOS ONE

**General Assessment:**

This manuscript addresses an important topic in pediatric radiation protection by proposing Diagnostic Reference Levels (DRLs) in interventional cardiology based on a large, multicenter French cohort. The topic is of high relevance given the increasing number of pediatric congenital heart disease interventions and the associated long-term risks of radiation exposure.

While the study is generally well-conceived and written, the manuscript will benefit from deeper methodological transparency, stronger discussion of implications, and standardization of terminology and units.

I have made some suggestions as follows:

**1. Abstract:**

1. The sentence “this study provides…” should be capitalized.

**2. Introduction:**

1. The introduction would benefit from a clearer definition of study objectives. Is the aim to *establish* new national DRLs, or *compare* existing practice to European standards?
2. If there is any prior work done on pediatric DRLs, from other countries (UK, Germany, NCRP), should be cited to situate this work within the broader literature.

**3. Materials and Methods:**

1. There is insufficient detail on the imaging systems used (e.g., frame rates, filtration, pulse width).
2. The method for constructing the DRL-weight curve is not adequately explained. Please describe the mathematical approach e.g., regression model type.
3. Can you please specify the weight groupings and their justification. Were these based on existing DRL recommendations e.g., ICRP 135?

**4. Results:**

1. Rationale for no significant variation in fluoroscopy time (FT) across procedures is unclear. This contradicts typical expectations.
2. Is it possible to increase the resolution of figures/graphs you have uploaded?
3. Ranges of DAP and FT should always be accompanied by units and clear specification e.g., Median DAP = 2.5 Gy·cm², 75th percentile = 3.9 Gy·cm².

**5. Discussion:**

1. Adding more literature in discussion, how do the proposed DRLs compare with those from the UK, Germany, or ICRP will make this part more valuable.
2. Address potential limitations related to inter-center variability in dose protocols, fluoroscopy time settings, operator experience, and patient complexity.

**6. Conclusion:**

1. A recommendation for future work (e.g., periodic updates, inclusion of newer technology) would strengthen the conclusion.

**7. Ethics and Data Sharing:**

1. The ethics approval date December 2024 is after data collection (2018–2020), which raises questions about retrospective authorization. Please clarify.
2. Can you please specify whether anonymized data can be shared upon request.
