# Supplementary material for: Diagnostic Reference Levels in pediatric interventional cardiology: A multicenter study by the French cohort in HARMONIC project
Source: PLoS One. 2025 Sep 17;20(9):e0332241. doi: 10.1371/journal.pone.0332241 (PMC12443263; doi:10.1371/journal.pone.0332241)
Supplement: Attachment — Submitted filename: Response-to-Reviewers.docx [file pone.0332241.s004.docx]

**Response to Reviewers**

**Manuscript reference: PONE-D-25-10735**

**Title: Diagnostic Reference Levels in pediatric interventional cardiology: a multicenter study by the French cohort in HARMONIC project**

Dear Prof ARNABJYOTI DEVA SARMA,

We express our gratitude for your response and your valuable input. Your feedback, particularly the reviewers’ comments, is greatly appreciated. Thank you very much for your time and valuable insights. Please find in this resubmission letter our point-by-point response to the reviewers’ comments. Based on their suggestions, we submit a revised manuscript in a marked-up copy that highlights changes made to the original version.

Kindest regards, yours sincerely,

Dr Bouchra Habib Geryes

**Reviewer 1’s Comments**

| **Comment** | **Author’s Response** |
| --- | --- |
| General Assessment:  This manuscript addresses an important topic in pediatric radiation protection by proposing Diagnostic Reference Levels (DRLs) in interventional cardiology based on a large, multicenter French cohort. The topic is of high relevance given the increasing number of pediatric congenital heart disease interventions and the associated long-term risks of radiation exposure.  While the study is generally well-conceived and written, the manuscript will benefit from deeper methodological transparency, stronger discussion of implications, and standardization of terminology and units.  I have made some suggestions as follows: | We thank the reviewer for the constructive general assessment and for acknowledging the relevance of our study. We appreciate the suggestions for improvement, which have been addressed in the revised manuscript using tracked changes. |
| 1. Abstract:  1. The sentence “this study provides…” should be capitalized. | Done |
| 2. Introduction:  1. The introduction would benefit from a clearer definition of study objectives. Is the aim to establish new national DRLs, or compare existing practice to European standards? | Thank you for your helpful comment. There is a general lack of comprehensive data on radiation doses in pediatric interventional cardiology both in France and internationally. This gap highlights the need for multicenter studies covering a broad range of IC procedures to develop more robust and widely applicable DRLs in this field. The primary objective of this study is to establish the first multicenter DRLs in France. These newly defined DRLs will serve as national benchmarks and also contribute internationally by addressing the existing data shortage in the literature.  The introduction has been revised to clearly state the study objectives: “This study aims to address the general lack of comprehensive data on radiation doses in pediatric interventional cardiology. Its primary objective is to establish the first multicenter DRLs in France for various pediatric IC procedure types and body-weight groups in France. The cohort includes patients ranging from newborns to young adults up to 18 years old, representing a broad spectrum of ages, weights, and CHD types. This multicenter approach enhances the generalizability of the findings and provides valuable insights into radiation practices and outcomes across diverse clinical settings. The newly proposed multicenter DRLs can serve as national benchmarks and contribute to filling the data gap in the literature, supporting the development of a solid basis for developing future national and international guidelines in pediatric IC practice.” |
| 2. If there is any prior work done on pediatric DRLs, from other countries (UK, Germany, NCRP), should be cited to situate this work within the broader literature. | Thank you for your valuable suggestion. As discussed in the manuscript, existing literature from countries such as the UK and Germany includes general pediatric DRLs (CT, fluoroscopy, radiography), but specific DRLs for pediatric interventional cardiology remain scarce and are often not well differentiated by procedure type. Most available studies tend to group all procedures into a single therapeutic category, which limits meaningful comparisons. Furthermore, ICRP Report 135 emphasizes the importance of establishing procedure-specific DRLs but does not provide specific reference values for pediatric interventional cardiology.  We conducted a thorough literature search on pediatric Diagnostic Reference Levels (DRLs) in interventional cardiology. In our discussion, we have included, to the best of our knowledge, all pertinent international studies reporting DRLs in this field, as summarized in Table 4, and we have highlighted how our results compare to and expand upon this limited but essential body of literature.  As part of the HARMONIC project, the collection of dosimetric data has been initiated in the participating European countries. However, this work is still ongoing, and no national results have been published to date. This has been added in the discussion paragraph: “This classification was first developed at the national level, following the specific protocol of the COCCINELLE study, and then harmonized at the European level in the Harmonic project. In this context, the collection of dosimetric data has been initiated in several participating European countries; however, this work is still in progress, and no national results have been published to date.” |
| 3. Materials and Methods:  1. There is insufficient detail on the imaging systems used (e.g., frame rates, filtration, pulse width). | Thank you for your comment. We agree that detailed technical parameters of the imaging systems, such as frame rates, filtration, and pulse width, may influence dose levels and are important considerations in dose optimization. However, these parameters were not systematically collected across all centers and could therefore not be included in our analysis. It is also important to note that these technical parameters generally vary according to the type of procedure and the child’s age and weight. Nevertheless, the DAP inherently reflects the combined influence of these parameters, including frame rate, filtration, pulse width, and source-to-image distance, etc.. For multicenter DRL studies, DAP remains an appropriate and robust dosimetric indicator.  We have acknowledged this point in the revised manuscript by adding it to the limitations section: “Third, detailed technical parameters of the imaging systems, such as frame rate, filtration, and pulse width, were not systematically collected across all centers and therefore could not be included in the analysis. These parameters typically vary depending on the procedure type as well as the patient’s age and weight. However, the DAP inherently reflects the combined influence of these parameters, making it a relevant and robust dose metric in this context. Despite this limitation, the multicenter nature of the study, along with the standardized procedure categorization and weight-based stratification, supports the clinical relevance of the proposed DRLs.”. |
| 2. The method for constructing the DRL-weight curve is not adequately explained. Please describe the mathematical approach e.g., regression model type. | DRL-weight curves illustrate how the DAP varies as a function of the body weight for each procedure type. To construct these curves, we used quantile regression to model the 50^th^ percentile (median) DAP values according to body weight. This method allowed us to estimate the slope coefficients for each procedure category, which are reported in Table 3, while the resulting DRL-weight curves are presented on a log-log scale in Fig 4.  The statistical analysis paragraph has been revised to include the following clarification: “Additionally, to address challenges in defining patient groups and the limited available data, the DRL curves proposed by the European Guidelines on Diagnostic Reference Levels for Pediatric Imaging (RP185) [32] and other studies [13,21] were investigated. This approach aimed to mitigate issues related to collecting a sufficient number of procedures from a limited number of centers within a reasonable timeframe.  Regarding the DRL-weight curves, based on DAP in function of the body weight, quantile regression method [13,18,24,34] was applied to find the median curves for each IC procedure category. Pearson’s correlation coefficient was used to evaluate the alignment between DRL-weight curve values and collected data.” |
| 3. Can you please specify the weight groupings and their justification. Were these based on existing DRL recommendations e.g., ICRP 135? | Thank you for your comment. The weight groupings used in our study are based primarily on existing recommendations, particularly those specified in the European RP185 report on Pediatric Diagnostic Reference Levels (PIDRL), as detailed in Table 7.1 on page 35. This is clearly specified in the data stratification paragraph of the manuscript. Using these standardized weight categories ensures comparability with other studies and alignment with established guidelines. |
| 4. Results:  1. Rationale for no significant variation in fluoroscopy time (FT) across procedures is unclear. This contradicts typical expectations. | Thank you for your comment. As mentioned in the manuscript, the very low Spearman correlation coefficient (ρ = -0.0036) suggests an absence of meaningful association between fluoroscopy time and patient body weight, whereas the correlation between DAP and weight was moderately positive (ρ = 0.57) as also observed in other studies [13,20]. The variations observed in DAP are probably more related to factors such as the extent of radiography use, source-to-detector distance, filtration, dose per image, or dose per frame rather than fluoroscopy time alone that primarily reflects procedural complexity. Moreover, categorizing procedures into specific groups helps reduce variability in fluoroscopy time. |
| 2. Is it possible to increase the resolution of figures/graphs you have uploaded? | The figures have been revised to address the various reviewers’ comments. They have been carefully checked and regenerated using the Preflight Analysis and Conversion Engine (PACE) digital diagnostic tool recommended by the editor. |
| 3. Ranges of DAP and FT should always be accompanied by units and clear specification e.g., Median DAP = 2.5 Gy·cm², 75th percentile = 3.9 Gy·cm². | Done |
| 5. Discussion:  1. Adding more literature in discussion, how do the proposed DRLs compare with those from the UK, Germany, or ICRP will make this part more valuable. | Thank you for your comment. This point has been addressed in our response to Comment 2. |
| 2. Address potential limitations related to inter-center variability in dose protocols, fluoroscopy time settings, operator experience, and patient complexity. | Thank you very much for this insightful comment. We fully agree and this point has been clarified in the revised Discussion section.: “The variability of DAP within the same procedure and weight subgroup may be partly explained by inter-center variability, reflecting differences in clinical practices, equipment, and protocols among centers. This inter-center variability highlights the diversity of real-world clinical settings captured by our multicenter study, and the proposed DRLs thus reflect a wide range of practices. An analysis of the impact of procedure complexity and operator experience on patient doses was not conducted but would be valuable in future investigations. Increasing the sample size would enhance the robustness and generalizability of the findings. Extending this work at the European level, using data from multiple centers across several countries as part of the HARMONIC project, will strengthen the findings and enable broader comparisons and insights across diverse healthcare settings.”. |
| 6. Conclusion:  1. A recommendation for future work (e.g., periodic updates, inclusion of newer technology) would strengthen the conclusion. | Thank you for this valuable suggestion. We have revised the conclusion to include these recommendations: “Future work should focus on periodic updates of these DRLs to reflect advances in imaging technology and evolving clinical practices, ensuring continuous improvement in radiation protection for pediatric patients.” |
| 7. Ethics and Data Sharing:  1. The ethics approval date December 2024 is after data collection (2018–2020), which raises questions about retrospective authorization. Please clarify. | The ethical approval was obtained in 2011 and updated in December 2024 because of legal modification of our research institute (IRSN became ASNR). This was already mentioned in the material and methods paragraph: “Ethical approval to conduct the study was granted by the French national data protection commission (Commission Nationale de l’Informatique et des Libertés), under approval number 911,112 on December 12, 2011 and deliberation N°2016-1103 on August 11, 2016, amended by deliberation N°2024-1240 on December 30, 2024.” |
| 2. Can you please specify whether anonymized data can be shared upon request. | A paragraph titled “Data Availability” has been added to the revised manuscript to clarify this point: “The ethical agreement does not allow data sharing without a data transfer agreement. Any request to access anonymized data should be addressed to our Data Protection Officer (donnees.personnelles@asnr.fr), who will assess the feasibility of sharing the data.” |

**Reviewer 2’s Comments**

| **Comment** | **Author’s Response** |
| --- | --- |
| Technical Soundness and Data Support:  The study is technically sound, and the conclusions drawn are well-supported by the experimental data provided. The rationale behind the research is clear, and the findings contribute meaningfully to the growing body of knowledge at the intersection of molecular biology and oncology.  Statistical Analysis:  The statistical analyses appear to have been performed appropriately and rigorously. The choice of statistical tests is suitable for the data type and research design, and the results are reported with sufficient detail and clarity.  Data Availability:  I appreciate that the authors have made all underlying data available. This transparency enhances the credibility of the findings and allows for future replication and further exploration by the research community.  Clarity and Language:  The manuscript is well-written and presented in clear, standard English. The structure is logical and facilitates comprehension, even for readers outside the immediate field of study. Minor editorial polishing could enhance readability further, but overall, the writing is of high quality. | We sincerely thank the reviewer for the positive and encouraging feedback. Improvements have been made in the revised manuscript using tracked changes to further enhance readability, as suggested. |

**Reviewer 3’s Comments**

| **Comment** | **Author’s Response** |
| --- | --- |
| Minor changes required in the figures:  Comment 1:  In the figure 2(A),2(B),2(C),2(D),2(E),2(F),2(G) and 3 the Range, Median Line, Mean and outliers are to be depicted in one corner. | Thank you very much for your suggestion. The median, IQR, and 95% IC values for each procedure and weight group are provided in detail in Table 2. Adding these values directly to each individual subplot in Figures 2 and 3 would risk overcrowding the figures and potentially reducing their readability, particularly given the number of weight subgroups. Furthermore, the axis scales and the boxplot representation allow readers to visually assess these values with reasonable accuracy. We believe that the current presentation strikes a balance between clarity and conciseness. |
| Comment 2:  In the Figure 4(A),4(B),4(C),4(D),4(E),4(F) and 4(G) Linear fit equation to be generated and paste in the figure. | Thank you for your comment. The figures have been updated accordingly, with the requested elements clearly shown. |

**Academic Editor comments**

| **Comment** | **Author’s Response** |
| --- | --- |
| Journal requirements:  When submitting your revision, we need you to address these additional requirements.  1. Please ensure that your manuscript meets PLOS ONE's style requirements, including those for file naming. | We thank the Academic Editor for the comment. We have carefully revised our manuscript to address all the reviewers’ comments. In addition, we have ensured that the manuscript complies with PLOS ONE’s style requirements. All figures were checked and corrected using the PACE digital diagnostic tool. All modifications in the revised manuscript are marked using tracked changes. |
| 2. We note that your Data Availability Statement is currently as follows: [All relevant data are within the manuscript and its Supporting Information files.]  Please confirm at this time whether or not your submission contains all raw data required to replicate the results of your study. Authors must share the “minimal data set” for their submission. PLOS defines the minimal data set to  consist of the data required to replicate all study findings reported in the article, as well as related metadata and methods (<https://journals.plos.org/plosone/s/data-availability#loc-minimal-data-set-definition>).  For example, authors should submit the following data:  - The values behind the means, standard deviations and other measures reported;  - The values used to build graphs;  - The points extracted from images for analysis.  Authors do not need to submit their entire data set if only a portion of the data was used in the reported study.  If your submission does not contain these data, please either upload them as Supporting Information files or deposit them to a stable, public repository and provide us with the relevant URLs, DOIs, or accession numbers. For a list of  recommended repositories, please see <https://journals.plos.org/plosone/s/recommended-repositories>.  If there are ethical or legal restrictions on sharing a de-identified data set, please explain them in detail (e.g., data contain potentially sensitive information, data are owned by a third-party organization, etc.) and who has imposed them (e.g., an ethics committee). Please also provide contact information for a data access committee, ethics committee, or other institutional body to which data requests may be sent. If data are owned by a third party, please indicate how others may request data access. | Ethical agreement does not allow sharing the data without a data transfer agreement. Any request to obtain anonymised data should be sent to our data protection officer (**donnees.personnelles@asnr.fr)**, who will examine the possibility of sharing these data. |
| 3. Please include a caption for figure 2A to 2G and figure 4A to 4G. | Done and the figures have been revised to address the various reviewers’ comments. |
