# Supplementary material for: Diagnostic Reference Levels in pediatric interventional cardiology: A multicenter study by the French cohort in HARMONIC project
Source: PLoS One. 2025 Sep 17;20(9):e0332241. doi: 10.1371/journal.pone.0332241 (PMC12443263; doi:10.1371/journal.pone.0332241)
Supplement: Attachment — Submitted filename: Reviewer comment.docx [file pone.0332241.s002.docx]

Comment 1 :

In the figure 2(A),2(B),2(C),2(D),2€,2(F),2(G) and 3 the Range,Median Line,Mean and outliers are to be depicted in one corner.

Comment 2:

In the Figure 4(A),4(B),4(C),4(D),4(E),4(F) and 4(G) Linear fit equation to be generated and paste in the figure.
